# “Chronic Disseminated Aspergillosis,” a Novel Fungal Immune Reconstitution Inflammatory Syndrome

**DOI:** 10.1093/ofid/ofaa175

**Published:** 2020-11-07

**Authors:** Annabelle Pourbaix, Romain Guery, Julie Bruneau, Estelle Blanc, Gregory Jouvion, Marthe Weinandt, Claire Rouzaud, Jérémy Lourenço, David Boutboul, Jean-Paul Mira, Alexandre Rouquette, Thierry Jo Molina, Marc Lecuit, Olivier Lortholary

**Affiliations:** 1 Paris University, Infectious Diseases and Tropical Medicine Department, Necker-Enfants Malades Hospital, AP-HP, Paris, France; 2 Pathology Department, Necker-Enfants Malades Hospital, AP-HP, Paris Descartes University, Paris, France; 3 Nuclear Medicine Department, Marie Lannelongue Hospital, Le Plessis-Robinson, France; 4 Pathophysiology of Pediatric Genetic Diseases, Sorbonne Université, INSERM, Assistance Publique Hôpitaux de Paris, Hôpital Armand-Trousseau, UF Génétique Moléculaire, Paris, France; 5 Experimental Neuropathology Unit, Institut Pasteur, Paris, France; 6 Visceral Surgery Department, Cochin Hospital, AP-HP, Paris Descartes University, Paris, France; 7 Clinical Immunopathology Department, St Louis Hospital, AP-HP, University of Paris, INSERM U967 HIPI, Paris, France; 8 Medical Intensive Care Department, Cochin Hospital, AP-HP, Paris Descartes University, Paris, France; 9 Pathology Department, Cochin Hospital, AP-HP, Paris Descartes University, Paris, France; 10 Biology of Infection Unit, Institut Pasteur, INSERM U1117, Paris, France; 11 Molecular Mycology Unit, Institut Pasteur, CNRS UMR2000, Paris, France

**Keywords:** aspergillosis, hepatosplenic, immune reconstitution inflammatory syndrome

## Abstract

We report a case of chronic hepatosplenic aspergillosis following immune reconstitution complicating colic aspergillosis in an AIDS patient with multicentric Castleman disease. Symptoms mimicked the clinical presentation of chronic disseminated candidiasis and responded to corticosteroid. This emerging entity enlarges the spectrum of fungal immune reconstitution inflammatory syndrome in the HIV setting.

Immune reconstitution inflammatory syndrome (IRIS) is defined as the clinical worsening or appearance of an infectious disease after reversal of immune deficiency. In the setting of HIV, it has been commonly described during the first months of antiretroviral therapy (ART) and associated with a wide range of opportunistic agents. Among fungal IRIS, cryptococcosis has been best described in HIV-infected patients and solid organ transplant recipients, whereas reports of IRIS associated with other fungi remain anecdotal [1]. We report here a new HIV-associated fungal IRIS, which we propose to name “chronic disseminated aspergillosis” given its similarities with chronic disseminated candidiasis [2, 3].

## CASE REPORT

A 39-year-old Caucasian man was admitted to the intensive care unit for multi-organ failure. He was diagnosed with HIV infection and HHV-8-associated multicentric Castleman disease with hemophagocytic syndrome confirmed by an excisional biopsy of an axillary lymphadenopathy and a bone marrow biopsy, associated with a Kaposi sarcoma of the foot. His CD4+ T-cell count was 13/mm^3^ (2%), and his HIV viral load was 28 979 copies/mL. He received intravenous etoposide (100 mg/m^2^/d for 2 days) and rituximab (375 mg/m^2^/wk for 2 weeks). His condition rapidly improved with resolution of hemophagocytosis.

Five days after etoposide infusion, he developed febrile neutropenia (day 0) (Supplementary Appendix 1) with septic shock requiring vasopressors and antibiotic therapy with meropenem and aminoglycoside. Caspofungin was added on day 3 for persistent fever. Bone marrow recovery occurred on day 4, and antiretroviral therapy was started with emtricitabine, dolutegravir, and enfuvirtide. On day 6, serum galactomannan antigen returned strongly positive (index value 9), and a culture of bronchial aspirate was positive for *Aspergillus fumigatus* without any suggestive feature of invasive pulmonary aspergillosis on thoracic computed tomography (CT). Caspofungin was switched to voriconazole to cover a possible invasive mold infection.

On day 8, a new septic shock with multi-organ failure occurred. An abdominal CT scan showed pneumoperitoneum. Laparotomy revealed fecal peritonitis and transmural colic necrosis requiring peritoneal toilet, subtotal colectomy with double-end ileostomy, sigmoidostomy, and cholecystectomy. Examination of the colectomy sample evidenced proven invasive aspergillosis with ulcerative, necrotizing, and hemorrhagic colitis containing fungal hyphae invading the digestive wall blood vessels (homogenous size, absence of pigmentation, acute angle branching and septation) with a positive labeling after anti-*Aspergillus* spp. immunohistochemistry (Supplementary Appendices 2 and 3). Caspofungin therapy was thus initiated on day 13, in combination with voriconazole because of the severity of illness. There was no radiological evidence of pulmonary, cerebral, or sinus aspergillosis. The patient’s condition gradually improved, apyrexia was obtained, and serum galactomannan antigen was negative on day 34. Combination therapy was switched to voriconazole alone after 13 days.

The patient presented an early immune reconstitution, with an increase in CD4+ T-cell count reaching 141/mm^3^ (13%) on day 16 (after 12 days of antiretroviral therapy) and an undetectable HIV viral load.

On day 25, he developed isolated fever without localizing signs or symptoms, elevated C-reactive protein serum levels, or eosinophilia (4, 600/mm^3^), and liver function tests were normal. An abdominal CT scan revealed multiple infracentimetric liver abscesses, and treatment with ceftriaxone and metronidazole was initiated. After 3 weeks of this empirical antibiotic therapy driven by what were considered pyogenic hepatic abscesses, abdominal magnetic resonance imaging showed no improvement, with multiple hepatosplenic lesions and a more localized lesion that appeared to be a peritoneal abscess in the left upper quadrant. A biopsy of this lesion was performed; microscopic examination revealed acute septate hyphae, fungal cultures were negative, and in-house real-time polymerase chain reaction (PCR) for *A. fumigatus* was positive (Supplementary Appendix 2). Histologic examination showed granulomas, necrosis, and acute septate hyphae (Supplementary Appendix 4). Serum galactomannan antigen remained negative. Diagnosis of hepatosplenic aspergillosis with HIV-associated IRIS triggered by antiretroviral therapy was retained. Antiretroviral therapy and voriconazole were continued, and the patient’s condition spontaneously improved with apyrexia, normal inflammatory blood parameters, and normal eosinophilic count on day 58.

**Figure 1.**
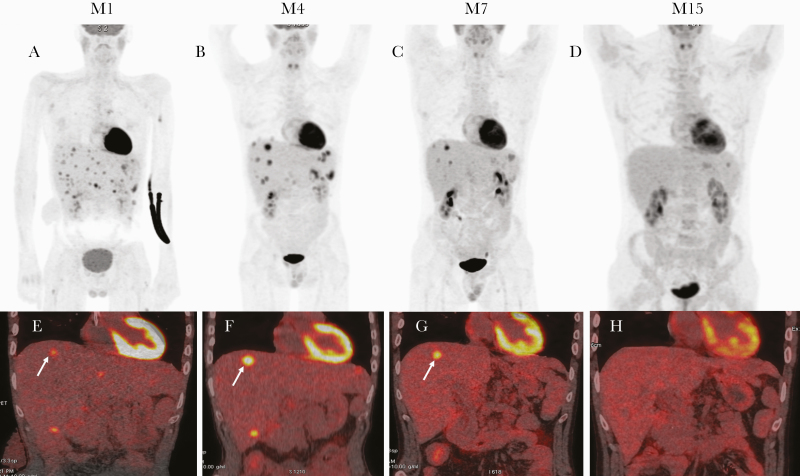
18-FDG positron emission tomography/computed tomography (PET/CT) during disease course. 18-FDG PET/CT scan maximum intensity projection (MIP) showed a metabolic modification with progression of several liver nodules despite the regression in numbers between month 1 (M1) (A) and month 4 (M4) (B), then decreased FDG uptake in the liver abscess and regression in uptake of numbers of lesions at month 7 (M7) (C) and complete regression at month 15 (M15) (D). Fused coronal PET and CT images show several abscesses with a target on the liver dome. In (E) (M1), the arrow indicates a liver node (SUV max, 6; metabolic tumor volume, 2.3 cm^3^). In (F), the arrow indicates an increase in FDG uptake in liver target (SUV max, 11.6; metabolic tumor volume, 4.5 cm^3^) at M4. G, Decreased metabolic activity (SUV max, 9.8; metabolic tumor volume, 2.5 cm^3^) at M7. H, No metabolic activity at M15.

After 4 months of voriconazole therapy, asthenia with weight loss appeared, with elevated C-reactive protein serum levels and anicteric cholestasis. The alkaline phosphatase serum level was 462 U/L (reference range, <120 U/L), and the gamma glutamyltransferase serum level was 920 U/L (reference range, <55 U/L). Galactomannan antigen in serum was negative. The patient’s HIV viral load had remained undetectable, and his CD4+ T-cell count was 204/mm^3^ (10%). 18-FDG positron emission tomography (PET)/CT showed increased hepatosplenic metabolism (Figure 1). There was no hemophagocytosis and no hypermetabolic lymph nodes. Histologic examination of a liver biopsy showed fibrosis, granulomas, and necrosis, without hyphae (Supplementary Appendix 4). Microscopic examination as well as bacterial, fungal, and mycobacterial cultures were negative. PCR for *A. fumigatus* was also negative. We concluded worsening of hepatosplenic aspergillosis related to an IRIS. Corticosteroid therapy was initiated at 0.5 mg/kg daily, and antifungal treatment was continued. Corticosteroid therapy was tapered down gradually over 2 months. Inflammatory blood parameters and liver function tests rapidly normalized. 18-FDG PET/CT showed improvement with partial metabolic response (Figure 1).

Seven months after relapse, the patient still presented immunovirologic response: undetectable HIV viral load and CD4+ T-cell count of 414/mm^3^ (13%). Liver function tests were normal, as well as inflammatory blood parameters and galactomannan antigen. At 15 months, 18-FDG PET/CT showed complete metabolic response (Figure 1).

## DISCUSSION

Here, we report the first case, to our knowledge, of what we propose to name “chronic disseminated aspergillosis” as an IRIS fungal syndrome occurring during HIV infection.

The patient originally presented with proven primary colic invasive aspergillosis, without evidence of pulmonary disease, as described by Eggimann et al., for whom the digestive tract is considered a potential portal of entry for *Aspergillus* spp. in immunocompromised patients [4]. Colic invasive aspergillosis was in our case characterized by transmural tissue necrosis with tissue invasion by septate hyphae and angioinvasion associated with elevated galactomannan antigen and positive immunohistochemistry with *Aspergillus* spp. antibody, fulfilling the 2008 definition criteria [5].

Our patient developed during immune reconstitution (neutrophil recovery on day 4 and CD4+ T-cell recovery >140/mm^3^ on day 16), symptoms strongly mimicking those presented by patients with chronic disseminated candidiasis: fever that fails to respond to antibiotics, increased alkaline phosphatase and C-reactive protein levels, hepatosplenic micro-abscesses on imaging, granulomatous lesions associated with fungal hyphae, but negative fungal cultures. Initially, clinical course was spontaneously favorable without any change in antifungal treatment. A relapse of IRIS occurred 4 months later, requiring corticosteroid therapy, with subsequent favorable outcomes.

Since the availability of highly active antiretroviral therapy (HAART), IRIS has been described as the paradoxical worsening of treated opportunistic infections or the unmasking of previously untreated infections, occurring classically when CD4+ T-cell counts rise and function improves. HIV-associated IRIS has been described for several fungal infections, such as cryptococcosis, *Pneumocystis jiroveci* pneumonia, histoplasmosis, candidiasis, and talaromycosis [1, 6–12]. Sambatakou et al. also reported a case of IRIS with invasive pulmonary aspergillosis in an end-stage HIV-infected patient [13].

Miceli et al. described pulmonary IRIS among patients with hematological malignancies and invasive pulmonary aspergillosis [14]. This entity is characterized by worsening of pulmonary symptoms and radiological lesions during neutrophil recovery, associated with a persistent microbiological response (eg, decrease in galactomannan index titers).

In hematological patients with profound and prolonged neutropenia, chronic disseminated candidiasis (CDC), also known as hepatosplenic candidiasis, is regarded as being due to an IRIS upon neutrophil recovery, favoring antigen-driven response [3]. Indeed, we recently evidenced an expansion of *Candida*-specific interferon-γ-producing T cells together with features of T-cell activation and systemic inflammation during CDC [2].

Invasive hepatosplenic aspergillosis without IRIS has been described in few cases in immunocompromised patients [15–17], but it has not been described in HIV-infected patients in historical papers [18], nor in a recent nation-based study [19] or an immunocompetent patient [20]. In our patient, we assume that initial colonization of the digestive tract with *Aspergillus* and subsequent colic invasive aspergillosis with high inoculum and transmural colic necrosis favored peritoneal dissemination, translocation to the bloodstream, and hematogenous spread via portal venous circulation, leading to occult hepatosplenic aspergillosis. Furthermore, hepatosplenic aspergillosis occurred when neutrophil and CD4+ T-cells started to rise and continued as immune reconstitution was going on. The patient developed peritoneal and hepatosplenic aspergillosis lesions almost 3 weeks after beginning treatment with voriconazole, with normal plasma concentrations and a microbial load control characterized by serum galactomannan antigen decrease. In addition, there was no evidence of new lesions of aspergillosis or other infectious processes in other organs. Fungal cultures remained negative, and subsequent improvement of the patient’s condition without treatment modifications or corticosteroid therapy supports the immune-related nature of the symptoms.

To conclude, this case supports the idea that independent of fungal species, colonization and primary invasive fungal infection of the digestive tract could be responsible for hepatosplenic IRIS in immunocompromised patients, including neutropenic AIDS patients with early immune restoration. Given the similarities with CDC, we propose to name this new entity “chronic disseminated aspergillosis” (CDA).

## Supplementary Material

ofaa175_suppl_Supplementary_Appendix
