# Supplementary material for: “Chronic Disseminated Aspergillosis,” a Novel Fungal Immune Reconstitution Inflammatory Syndrome
Source: Open Forum Infect Dis. 2020 Nov 7;7(11):ofaa175. doi: 10.1093/ofid/ofaa175 (PMC7651489; doi:10.1093/ofid/ofaa175)
Supplement: ofaa175_suppl_Supplementary_Appendix [file ofaa175_suppl_supplementary_appendix.docx]

**APPENDIX**

**S1 Appendix. Timeline.**

**S2 Appendix. Methods.**

**S3 Appendix. Histological examination of large intestinal resection specimens.**

**S4 Appendix. Histological examination of peritoneal abscess and hepatic granuloma.**

**S1 Appendix. Timeline**

day -25 : first symptoms

day -11 : admission in ICU for multi organ failure

day -5 : etoposide and rituximab infusion for multicentric Castleman disease and hemophagocytic syndrome

day 0 : febrile neutropenia

day 4 : bone marrow recovery and initiation of ART

day 6 : VCZ

day 8 : septic shock and angioinvasive colic aspergillosis

day 16 : increase in CD4+ T-cell count (141/mm3), undetectable HIV viral load

day 25 : first symptoms of CDA, continuation  of ART + VCZ

day 34 : negative serum galactomannan antigen

day 58 : disparition of symptoms of CDA (ART + VCZ)

day 120 : relapse of CDA treated with corticosteroids (combined with VCZ and ART)

day 180 : discontinuation of corticosteroids

day 210 : no evidence of clinical relapse of CDA

ART: Antiretroviral therapy

CDA: Chronic disseminated aspergillosis

ICU: Intensive care unit

VCZ: Voriconazole

**S2 Appendix. Methods**

*Immunohistochemistry.*

Anti-*Aspergillus* immunohistochemistry analysis was carried out using a primary mouse monoclonal antibody (GeneTex GTX42724 ; WF-AF-1, dilution 1:50), designed for research applications, recognizing members of the *Aspergillus* spp. including *A. fumigatus, A. flavus* and *A. niger*.

*RT-PCR assay.*

A. fumigatus DNA was amplified by real-time PCR with a Thermocycler/ABI Prism 7300 sequence detector (Applied Biosystems) targeting a 67-bp DNA fragment specific to the multicopy gene encoding the 28S rRNA of A. fumigatus, as previously described.^4^ Positivity of sample was considered only when the crossing point value was ≤41 cycles.


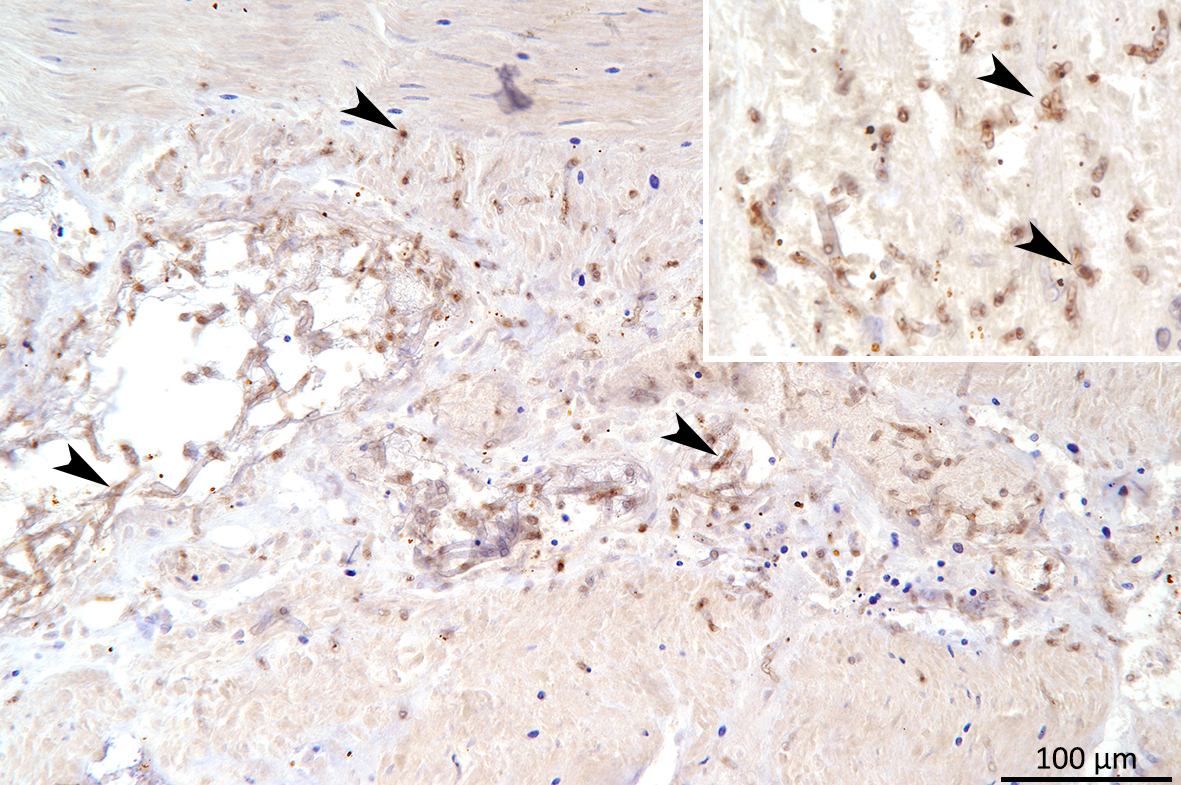


**S3 Appendix. Histological examination of large intestinal resection specimens**

Subacute and necrotizing ulcerative colitis, with intra-lesional hyaline hyphae, positively labeled after anti-*Aspergillus* immunohistochemistry (black arrowheads: labeled hyphae).


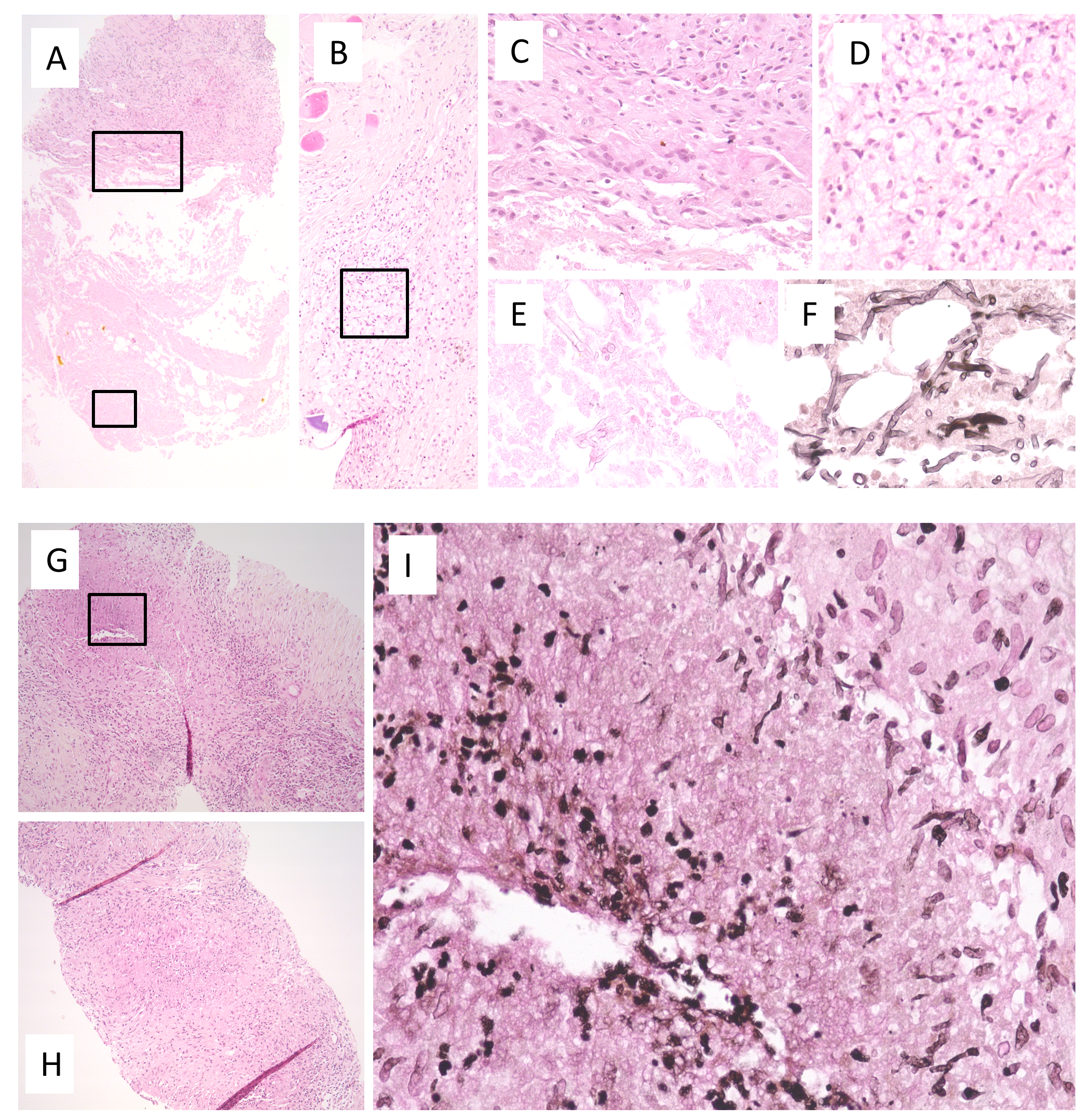


**S4 Appendix. Histological examination of peritoneal abscess and hepatic granuloma**

*Peritoneal abscess biopsies*. A and B: inflammatory and necrotic infiltration of a fibrous connective tissue. C: inset picture (A) showing multinucleated giant cells. D: inset picture (A) showing foamy macrophages. E (Hematoxylin Eosin Saffron staining) and F (Grocott staining): septated and branched mycelial filaments.

*Liver biopsy*. G and H: large and necrotic granuloma. I: inset picture (A) showing granuloma without mycelial filaments at the Grocott staining.
